# Canadian Sarcoma GroupWorkshop Sarcomas: Molecular Markers to Therapeutics, 26–27 February 2000,Toronto, Canada

**DOI:** 10.1155/S1357714X00000128

**Published:** 2000-03


*Sarcoma* (2000) **4**, 67–73

MEETING REPORT

### Canadian Sarcoma Group Workshop Sarcomas: Molecular Markers to Therapeutics, 26–27 February 2000, Toronto, Canada

**Organizing Committee**:

Vivien Bramwell (Chair)

Irene Andrulis
Robert Bell
Elizabeth Eisenhauer
Victor Fornasier
Rita Kandel
Brian O'Sullivan
Walley Temple
Robert Turcotte
Jay Wunder

Address for reprints: Dr Vivien Bramwell, London Regional Cancer Centre, 790 Commissioners Road East, London, Ontario, Canada N6A 4L6, Tel (519) 685-8640; Fax (519) 685-8624

1357-714X print/1369-1643 online/00/010067-07 © 2000 Taylor & Francis Ltd

### INTRODUCTION

The Canadian Sarcoma Group (CSG) held its third national workshop "Sarcomas: Molecular Markers to Therapeutics", at the Colony Hotel, Toronto, 26-27 February 2000. The meeting was attended by approximately 50 speakers and participants (Appendix 1) representing a broad range of disciplines including scientists, medical and radiation oncologists, pathologists, radiologists and community representatives. The format of the Workshop encouraged audience participation and there were lively discussions within each of the four sessions.

The overall objectives of the Workshop were to do the groundwork for a five year program of scientific and clinical trials activity for the CSG. Specific aims of each session are outlined in their titles.

### SESSION 1

### DEVELOPMENT OF NEW MOLECULAR MARKERS CORRELATING WITH CLINICAL OUTCOMES IN SOFT TISSUE SARCOMA (STS)

### MODERATOR: *IRENE ANDRULIS*

### SARCOMA BIOLOGY - FROM GENETICS TO SIGNAL TRANSDUCTION

***Poul Sorensen, Vancouver***

Dr Sorensen reviewed some of his work demonstrating the presence of a translocation12,15 in congenital fibrosarcoma, which distinguishes it from fibromatosis and low-grade adult fibrosarcomas. This translocation causes the formation of chimeric oncoprotein, ETV6-NTRK3, which is a tyrosine kinase. Expression of this protein in NIH3T3 cells is oncogenic, and transformation requires both the ETV6 helix-loop-helix dimerization domain and the NTRK3 protein tyrosine kinase domain. Dr. Sorensen has created a mouse model, showing that incorporation of this oncoprotein in 3T3 cells renders them tumorigenic.

### MICRO-ARRAY TECHNIQUES IN SARCOMA

***Jay Wunder, Toronto***

Dr Wunder explained the potential of micro-array techniques in assessing global patterns of gene expression. Micro-array analysis offers the potential to simultaneously analyze mRNA levels of hundreds or thousands of genes. One of the goals is to identify "genetic profiles" or "signature patterns" of gene expression for specific tumors that might help to better classify them, predict outcome and response to therapy. This might also provide new gene targets for therapy. He briefly described an NIH Consortium grant application involving six centres including Toronto, brought together under the umbrella of the Connective Tissue Oncology Society. If funded, this grant would provide core facilities for tissue banks, pathology review, and bioinformatics and would include three main scientific projects examining the molecular profiles of liposarcoma, leiomyosarcoma/gastrointestinal stromal tumors (GIST) and malignant fibrous histiocytoma. He described some of the proposed Toronto-Rizzoli Institute work on liposarcoma, and finally outlined some of the challenges that arise in using these techniques.

### BIOLOGIC RESPONSE MODIFIERS IN RHABDOMYOSARCOMA

***David Malkin, Toronto***

Dr Malkin discussed the childhood rhabdomyosarcomas which share a common defect in myogenic differentiation. MyoD is expressed but is non-functional in tumors. Embryonal and alveolar types of rhabdomyosarcoma each show different translocations. He described some of his scientific work on the characteristics of MyoD. He went on to describe a series of experiments using retinoic acid, which is a differentiating agent in acute promyelocytic leukemia and neuroblastoma. He has shown that retinoic acid can cause growth arrest and morphological differentiation in some rhabdomyosarcoma cell lines. He plans a further series of experiments which he hopes will lead into some parallel clinical studies.

### MOLECULAR AND CYTOGENETIC APPROACHES TO IMPROVED OUTCOME ASSESSMENT IN SARCOMAS

***Jeremy Squire, Toronto***

Ewing's sarcoma (ES) is characterized by chromosomal rearrangements involving 22q12 which alter the EWS gene. In addition to these consistent aberrations, both numerical and structural aberrations have been reported: namely gains of chromosomes 8 and 12, the unbalanced translocation t(1;16), and deletions at the short arm of chromosome 1. To evaluate the frequency and to study the prognostic implications of some of these aberrations in children, Dr. Squire's group analyzed tumor specimens from 26 ES pediatric patients by classical cytogenetics and/or interphase fluorescence in situ hybridization (FISH) and compared these data with clinical parameters by interphase FISH analysis. Gains of chromosomes 8 and 12 were detected in 48% (10/21) and 38% (6/16) of the tumors respectively, and this was not significant with outcome. Statistical analysis revealed that the presence of a complex karyotype (ie. two or more structurally rearranged chromosomes) was associated with an unfavourable outcome when compared to ES tumors with simpler karyotypes ($p=0.0034$ was an independent prognostic value as an unfavourable marker).

### EXPERIMENTAL STUDIES OF THE EFFECTS OF RADIATION ON WOUND HEALING AND FIBROSIS

***Richard Hill, Toronto***

Combined radiation and surgery has proven to be highly successful in producing local control of several malignancies including STS. However, substantial long term morbidity may occur as a result of two complications: poor wound healing or post-radiation fibrosis. The NCIC-CTG/CSG SR2 trial demonstrated that STS patients receiving preoperative radiation had a 35% risk of developing wound healing complications. Tissue fibroblasts play an important role in wound healing as well as the development of fibrosis. Dr. Hill's group has shown that poor wound healing in rats caused by preoperative radiation can be partially reversed by transplanted autogenous fibroblasts but that this effect is lost if these fibroblasts had been previously irradiated. Dr. Hill is currently examining the radiosensitivity of skin fibroblasts from patients treated with preoperative irradiation for STS. Five of the 12 patients studied to date demonstrated wound healing complications. A clonogenic assay, with irradiation given at a low dose rate (LDR - 2.5 cGy/min), is used and for most patients at least two separate isolates of fibroblasts from their biopsies have been studied to determine the consistency of the measurements. The fibroblasts were characterized into three groups (sensitive, intermediate and normal) in relation to their radiosensitivity (dose required to give 1% survival). For the patients with wound healing problems 60% (3/5) had sensitive or intermediate sensitivity fibroblast vs 28 % (2/7) for patients without wound healing problems. A further 12 samples from a population of patients treated with preoperative irradiation for STS (7 with wound healing complications and 5 without) are under study. A study of TGF beta levels has been initiated since this cytokine plays an important role in wound healing and recent studies have reported that plasma levels correlate with the development of radiation-induced fibrosis.

### PANEL DISCUSSION WITH AUDIENCE PARTICIPATION

The first part of the discussion focused on molecular mechanisms that might be targets for therapy, including IGF-1, IGF-2, p53, MDR, and some of the techniques that might be used to detect alterations. Micro-array techniques may be able to identify additional targets, but issues such as quality of tissue and the influence of heterogeneity within the tumor, or hypoxia were also emphasized. In response to a question as to whether any markers could currently be used prospectively in adult STS, Dr Andrulis emphasized the need for proper epidemiological studies such as those that had been performed in breast cancer. Dr Malkin commented on the fact that, in U.S. pediatric trials, 23 gene/proteins are being analyzed by multiple laboratories. Samples are obtained from all patients entered into pediatric clinical trials through the Childrens Oncology Group. There was some additional discussion around prediction of tumor radiosensitivity.

The discussion then focused on the CSG tumor bank/database. In response to a query about how representative this might be for adult STS across the country, it was acknowledged that there is a bias to extremity sarcomas and primary tumors because of the specialties of the contributing surgeons. Dr Bell commented that the relative numbers could be compared with those recorded in the Ontario Cancer Registry. Dr O'Sullivan pointed out that although the project could be broadened by involving other surgeons, there may be logistic issues.

With respect to tumor banks, Dr Sorensen commented that handling of specimens may be important and advocated looking into new methods of culture, as well as freezing specimens. He felt it would be useful to have a bank of mouse human tumor xenograft models in which one could examine therapeutic manoeuvres. There was some discussion on the various mouse models that might be useful in this setting.

The final segment of the discussion focused on the best use of the CSG tumor bank/database to improve treatments. Dr Eisenhauer pointed out this issue would be discussed in the afternoon session. Dr Andrulis emphasized the importance of the first step of identifying suitable targets, and it was acknowledged the studies will be driven by the expertise of individual Canadian investigators and the types of material available in the bank. Dr Bell pointed out that this meeting was timely as it is only now that the CSG has a useful tumor bank with more than 800 specimens and follow-up clinical data. There was some additional discussion around technical issues relating to the bank.

### SESSION 2

### INVESTIGATIONAL NEW DRUG OPPORTUNITIES IN SOFT TISSUE SARCOMA

### MODERATOR: *VIVIEN BRAMWELL*

### NEW DRUGS OF POTENTIAL INTEREST IN SARCOMAS - EUROPEAN PERSPECTIVE

***Will Steward, Leicester, UK***

Dr Steward noted that the EORTC has performed two phase II studies/year for 20 years examining new agents, new schedules or modified doses of various drugs, but despite all this activity there remain only two active drugs in adult STS. There is a real need to maintain enthusiasm to continue investigational new drug studies. The EORTC will conduct a phase II study of irinotecan in GIST. Ectinascidin (ET-743), a compound derived from a marine organism that has broad activity in animal models including sarcoma, is in phase II study by the EORTC. Dr Steward described a number of interesting drugs under development in the UK. Most of these are in phase I evaluation with phase II studies planned in STS. An anthraquinone di-N-oxide (AQ4N), is a topo-isomerase II inhibitor. It enhances the effects of cisplatin and cyclophosphamide, and has synergy with radiotherapy. BBR 3464 is a novel trinuclear platinum compound. Combretastatin A4 phosphate shows potent selective toxicity for tumor associated endothelium. Finally, he described PTK 787 an aminophenothalazine which causes dose dependent inhibition of VEGF-R tyosine kinase and PDGF-R tyrosine kinase. This also is in phase I study, with associated dynamic MR imaging of tumor blood flow. Phase II studies are planned in soft tissue and osteosarcomas, which are high expressors of VEGF.

### TARGETING PEDIATRIC SARCOMAS FOR IMMUNE THERAPY

***Crystal Mackall, NCI, US***

Unfortunately Dr Mackall, despite valiant efforts, was unable to get to Toronto because of fog at Toronto Airport. The group hopes to have an opportunity to hear her talk at another time.

### NCIC-CTG INVESTIGATIONAL NEW DRUG PROGRAM

***Elizabeth Eisenhauer, Kingston***

Dr Eisenhauer described the NCIC-CTG IND program goals and history, and reviewed the activity of the Sarcoma Disease Site Committee during the 1980s/1990s, including studies of mitoxantrone, trimetrexate, 10-EDAM, topotecan and docetaxel. She emphasized that the way of the future is novel targeted agents and that the rationale for their use in sarcomas should be driven by molecular abnormalities of sarcomas. An example of a target is the Philadelphia chromosome in CML in which a t(9:22) translocation creates Bcl-Abl. The protein product of this translocation is a tyrosine kinase which is of fundamental importance in inducing malignancy. ST1 571 is an oral inhibitor of Bcl-Abl tyrosine kinase and causes a dose-related response in CML. Several sarcomas, such as Ewing's, rhabdomyosarcoma, myxoid liposarcoma, clear cell sarcoma, and synovial sarcoma, show specific translocations. Given their rarity it will be a challenge to develop designer drugs for sarcomas alone as it would not be a worthwhile financial investment for pharmaceutical companies. An alternative would be to target general signalling pathway abnormalities such as HER2/neu or molecular alterations such as mutations of p53, cyclin dependent kinases, and MDM2. She closed by discussing the rationale for studying GI checkpoint arrest compounds such as flavopiridol, which is about to enter phase II study by the NCIC-CTG/CSG.

### BIOLOGICAL THERAPIES IN NEUROFIBROMATOSIS-1 ASSOCIATED TUMORS

***Ab Guha, Toronto***

Dr Guha commented that neurofibromatosis-1 is the most common inherited genetic abnormality predisposing to cancer in humans. NF-1 associated peripheral nerve tumors includes large plexiform neurofibromas with a 3-5% incidence of malignant conversion to a neurofibrosarcoma. NF-1 occurs in 1/4000 live births and is autosomal dominantly transmitted with 100% penetrance, but variable and non-predictable expression. Half of the cases do not have a positive family history, representing de-novo germline mutations. The NF-1 gene is carried on chromosome 17, with one of the main functions of the encoded protein neurofibromin is to inactivate *ras*, a major signaling molecule. Therapies aimed at inhibiting *ras* activity are therefore under investigation, with agents such as farnesyl-transferase inhibitors (FTI). In addition, neurofibromas and neurofibrosarcomas are very well vascularized, with increased expression of the angiogenic growth factor Vascular Endothelial Growth Factor (VEGF). Small molecule inhibitors of the VEGF receptor, such as SU5416 (SUGEN Inc), have been demonstrated to be efficacious in inhibiting overall growth of human NF-1 neurofibrosarcomas in xenograft models, and similar anti-angiogenic strategies may be useful in this metastasizing malignant tumor. Of interest, FTIs not only decrease proliferation of transformed cells, but also decrease VEGF secretion, hence may have mitogenic and anti-angiogenic activities in solid tumors such as neurofibrosarcomas.

### AGGRESSIVE FIBROMATOSIS: LESSONS FROM DEVELOPMENTAL BIOLOGY AND IMPLICATIONS FOR TREATMENT

***Ben Alman, Toronto***

Dr Alman noted that aggressive fibromatosis had been associated with trisomy 3, trisomy 7 and deletion of the long arm of chromosome 5. It is associated with familial adenomatous polyposis coli, which also shows deletion of the long arm of chromosome 5. He described some of his studies of beta-catenin, which is a gene needed for the development of the wings of the drosophilia. In the human beta-catenin binds to cadherin. These studies have shown increased expression of both beta-catenin and COX-2 in fibromatosis. Various COX-2 inhibitors, such as sulindac, indomethacin and MercDFU do lead to decreased proliferation of cultures of aggressive fibromatosis, but also normal fibroblasts, and therefore the drugs are not selective.

### PANEL DISCUSSION WITH AUDIENCE PARTICIPATION

The initial part of the discussion focused on whether it would be possible to develop drugs that are specific for sarcomas or whether it is more likely that drugs developed for other tumors would be tested in sarcomas. It was agreed that the latter is more likely and that even compounds that may target specific points in the cell cycle could have more wide ranging effects. Dr Wong questioned the possibility of combination chemotherapy using novel agents. Dr Eisenhauer felt that this would be difficult until each of these agents had been properly evaluated. She felt there was more potential for combinations of novel agents with standard chemotherapy agents.

There was some discussion around the importance of rapid recruitment of patients to phase II trials in Canada and agreement that a Web site to advertise studies would be useful. In response to a question about availability of compounds in Europe, Will Steward outlined the benefits of the Cancer Research Campaign (CRC) formulation unit that had been developed in the UK. Elizabeth Eisenhauer commented that there was an Ontario translational research group that was looking into the possibility of liaisions with the CRC formulation unit. Dr Eisenhauer once again emphasized that there are many selectively targetted agents but it is important to identify molecular targets in STS. There was further discussion around the difficulties of obtaining interesting compounds, as most of these are now controlled by major pharmaceutical companies more interested in evaluating them in common tumor types.

### SESSION 3

### OPPORTUNITIES TO IMPROVE THE THERAPEUTIC RATIO OF LOCOREGIONAL MANAGEMENT IN EXTREMITY AND RETROPERITONEAL SOFT TISSUE SARCOMAS

### MODERATOR: *ROBERT BELL*

### WHO NEEDS RADIOTHERAPY AFTER RESECTION? EXPERIENCE OF THE SCANDINAVIAN SARCOMA GROUP

***Henrik Bauer, Stockholm, Sweden***

Dr Bauer addressed this topic by using features of Scandinavian sarcoma care, results from databases and previous studies, results from the Scandinavian Sarcoma Group (SSG) register, a review of the consequences of local recurrence, and of the local recurrence rates dependent on site and grade. He concluded that local recurrence is strongly associated with metastases, but is seldom the cause, and survival will not be increased by improvements in local treatment. Historically approximately 20% of patients were given radiotherapy but there has been an increaed use of radiotherapy within the SSG during the last couple of years.

He would recommend radiotherapy in certain situations, specifically 1) subcutaneous lesion if recurrence might lead to amputation (arm, lower leg, foot), 2) low-grade, deep lesions after marginal margins, 3) high-grade, deep lesions after marginal or wide margins. In contrast he felt that certain groups did not need radiotherapy: 1) subcutaneous lesions of trunk and thigh, 2) low-grade, deep lesions after wide margins, 3) high-grade, deep lesions after compartmental margin. He suggested that the consequences of this would be that 60% of all STS patients would have radiotherapy and local recurrence rate might drop from 20% to 10%. He posed the question as to whether the gain in local control was worth the cost and morbidity of radiotherapy.

### RADIATION DOSE/SCHEDULING ISSUES

***Martin Robinson, Sheffield, UK***

Dr Robinson agreed with Dr Bauer that local recurrence has little impact on survival, as recurrence is probably related to bad biology. He provided an overview of the various techniques in radiotherapy, including conformal radiotherapy, brachytherapy, intraoperative radiotherapy, neutron therapy, limb perfusion with radiotherapy, preoperative vs postoperative treatment, hyperfractionation, dose issues, re-irradiation and planning IMRT.

### LOCOREGIONAL CONTROL - CANADIAN (NCIC-CTG SR2) AND TORONTO EXPERIENCE

***Brian O'Sullivan, Toronto***

In the CSG SR2 study, 190 adult patients were randomized in a phase III trial comparing preoperative and postoperative radiotherapy in extremity STS. The key result was that the wound healing complications were significantly higher in the group receiving preoperative radiotherapy (31/88 patients, 35%) compared to postoperative radiotherapy (16/94 patients, 17% - p=0.01). A retrospective study at Princess Margaret Hospital (PMH), showed that the sequencing of radiotherapy influenced the radiation field size. A similar analysis of patients entered on SR2 showed that the field size was 50% greater for those receiving postoperative radiotherapy, and there were differences in the exposure of joints to radiotherapy between the preoperative and postoperative groups. Dr O'Sullivan also described a retrospective study using existing PMH databases, from 1975 to 1996, to determine whether local control and the use of adjuvant chemotherapy influenced distant recurrence and survival. Two groups were defined - a recent era where the treatment was structured but without chemotherapy (386 patients), and a previous era in which treatment was less structured but included adjuvant chemotherapy (206 patients). The endpoint was metastasis free survival (MFS). A much higher rate of local control was shown in the later era group (p=0.0001) but no difference in MFS (p=0.56). Interestingly, in multivariate analysis, the use of adjuvant chemotherapy was associated with a significantly better MFS and the achievement of local control had a similar effect, when the influence of all variables was controlled for in the prognostic model for the whole 20 year cohort.

### FUNCTIONAL AND QUALITY OF LIFE OUTCOMES IN SOFT TISSUE SARCOMA

***Aileen Davis - given by Bob Bell, Toronto***

This paper described preliminary results from the SR2 study using two functional outcome scales (MSTS and TESS) and a generic health status measure (SF36). Within the trial more than 90% of patients completed assessments at all time points. The general findings were that functional outcomes were decreased at randomization, and functional outcome/*quality of life declined until about 12 weeks post surgery but returned* to near normal by one year. The TESS and MSTS scores were lower at six weeks post surgery for the preoperative radiotherapy group. If the patient experienced wound healing complications, regardless of their treatment group, there were detrimental effects in all scores lasting to one year, and it affected the quality of life. The general interpretation of these data was that it was important to select the radiotherapy based on local anatomic factors.

### NEW STRATEGIES IN THE MANAGEMENT OF RETROPERITONEAL SARCOMAS

***Carol Swallow, Toronto***

Dr Swallow reviewed some of the problems arising when treating retroperitoneal sarcomas. She then went on to describe a Toronto study involving preoperative radiotherapy followed by radical resection ± brachytherapy. Patients with primary or recurrent retroperitoneal tumors, without metastasis, that were felt to be resectable, were included. Of 56 patients screened in 3½ years 39 were entered. Most of them were liposarcomas, and 50% were high-grade. 29 underwent total resection with many radical resections performed. 26 received preoperative radiotherapy, and 21 brachytherapy. Late toxicity was acceptable, with one perioperative death. However, an unexpected complication of acute duodenitis suggested that brachytherapy was not suitable for the upper abdomen. Three year survival was 60%.

### SURGICAL MARGINS: PAST, PRESENT AND FUTURE

***Robert Turcotte, Montreal***

Dr Turcotte started with a discussion of the historical definitions of margins and pointed out the lack of consensus of what constitutes a wide margin. A 2 cm of normal tissue surrounding the tumor is generally called wide by surgeons whereas pathologists would more readily call wide a margin of 1 cm. With the more routine use of radiation therapy in STS, studies have reported that margin either positive or negative is the important factor affecting local recurrence rate. Stokle has reported on a consensus between pathologists and surgeons that has resulted in R0: negative margin, R1: micro-positive margin, and R2: macroscopic residual. He also discussed the issue of avoiding biopsy as proposed by the Scandinavian Sarcoma Group but felt that, without a well designed study, the medicolegal issues were too important in North America to perform an extensive procedure without a confirmed diagnosis, and that neoadjuvant treatments could not be used with this approach. He suggested new directions which included MRI assessment of local invasiveness of the tumor and evaluation of the response to neoadjuvant treatments, better identification of metastatic patient with PET-scans and molecular probes, better understanding of tumor behavior through cytogenetics and molecular biopsy and the use of adjuvant treatments that might reduce the need for large margins.

### PANEL DISCUSSION WITH AUDIENCE PARTICIPATION

Questions focused around whether the size of the margin mattered. Dr O'Sullivan felt that this was less important with radical radiotherapy. Techniques of biopsy were discussed. Dr Bell felt that there was no clear evidence that fine needle biopsy increased local recurrence rate. Dr Swallow commented that for the retroperitoneal tumors open biopsy sometimes did not achieve a diagnosis because the ideal area was not necessarily biopsied, whereas under radiologic control it might be possible to take a biopsy from a more appropriate area. With respect to margins, Dr Bell felt that information was lost if margins were defined as positive or negative. He emphasized the need to measure margins. There was some discussion around the difficulties of grading tumors, and in using this information, because of the heterogeneity of tumors. In general, it was felt that high-grade tumors were more prone to local recurrence and most of these should receive radiotherapy, although other factors should also be taken into consideration.

### SESSION 4

### FUTURE OF THE CANADIAN SARCOMA GROUP

### INTERDISCIPLINARY HEALTH RESEARCH TEAM IN MUSCULOSKELETAL NEOPLASIA

***Robert Bell, Toronto***

Dr Bell described a Letter of Intent that he had submitted, as team leader, to a new federal program that offers grants for Interdisciplinary Health Research Teams (IHRT). If funded, this team would involve three main centres - Toronto, Vancouver and Montreal - with several investigators from each centre. It would also have as a core the facilities of the Canadian Sarcoma Group tumor bank and database, as well as local databases. It would focus on four projects - molecular profiles of sarcoma using micro-array technology, developmental pathways in sarcoma, radiation effects on fibroblast function, and disability evaluation in sarcoma treatment.

### RELATIONSHIP OF CSG WITH SARCOMA DISEASE SITE COMMITTEE OF NCIC-CTG

***Elizabeth Eisenhauer, Kingston***

Dr Eisenhauer reviewed the mandate and structure of NCIC-CTG. She described the selection of disease site committees, their chairs and executives. She then outlined the procedures necessary for development of phase II and III clinical trials through the Clinical Trials Group.

### CANADIAN SARCOMA GROUP - WHERE ARE WE GOING?

***Vivien Bramwell, London, UK***

Dr Bramwell briefly reviewed the history and activities of the CSG, which was formed in 1985 as a multidisciplinary group with the aims to conduct, develop, coordinate and stimulate research in the field of sarcomas. The Canadian Soft Tissue Sarcoma Tumor Bank/Correlative Clinical Database has accrued tumors from 712 patients for a total of 810 tumors in a seven year period. She then presented analysis of how well the CSG group had addressed the various aims. The objective of planning and executing clinical research studies had been addressed by several NCIC-CTG/CSG phase II and III clinical trials in adult STS. The aim of promoting experimental studies in the etiology, diagnosis, pathogenesis and evolution of sarcomas, has been addressed indirectly, in facilitating interactions between various scientists and establishing the tumor bank/ database. The third aim, to develop standard criteria for assigning histological subtype and grade and to assess and apply new research techniques to the classification of sarcomas, has not been addressed, mainly because of limited resources. The fourth aim was, in each of the main therapeutic areas, to develop guidelines for optimal management. Although the results of a survey of current practice was published in 1988, and a symposium was held at the Royal College of Physicians and Surgeons of Canada also in 1988, much of the recent activity with respect to guidelines is focused on a provincial basis through Cancer Care Ontario Practice Guidelines Initiative Sarcoma Group, and also guidelines developed in British Columbia. The fifth aim of collaboration, liaison with other multicentre cooperative groups and remaining informed about current international research had been fulfilled by regular attendance of Executive Committee members at meetings of the Connective Tissue Oncology Society (CTOS), the Intergroup initiative of the National Cancer Institute. Also, the CSG has performed Intergroup studies with the European Osteosarcoma Intergroup and the EORTC.

Looking to the future, Dr Bramwell posed the following questions:

1. Does the current structure facilitate input from members?
2. Does the structure cover the diverse interests in sarcoma and development of ideas/projects?
3. Does the structure allow us to move in new directions?

She suggested there might be some options regarding the structure of the CSG. She compared the benefits and problems of each structure.

1. Maintain the current structure and activities
2. Dues paying/professional association (similar to CTOS)
3. Further harmonize with NCIC-CTG Sarcoma Disease Site Group

### DISCUSSION

There was very little enthusiasm for a move to a dues paying organization modelled on CTOS. The number of sarcoma specialists is too small to make this a realistic proposition. Dr Bell felt that over the years there had been a natural evolution of the CSG structure and functions and that the group is addressing many of the important issues. There was a prolonged discussion about the number of meetings that might be held by the CSG, how these might be funded, and what the content might be. Some of the clinicians favoured educational review sessions but others felt that this might be better done at other meetings, particularly the CTOS meetings which have a strong educational component. However, concerns were expressed about the expense and difficulty of getting to many of these meetings. There was some concern about the lack of regional infrastructure of the CSG and about communications. A number of suggestions included the importance of development of a Web page and also a more structured planning function for trial development. Dr Eisenhauer suggested that, like other disease site committees of NCIC-CTG, it would be important to have small subgroups of 2-3 people to work up ideas for possible studies. Discussion among these groups could take place by email. Three suggested areas for ideas generations were locoregional control, molecular studies, systemic treatment.

The importance of capturing as much data as possible from the Canadian sarcoma population was emphasized. Dr Bell felt that if the IHRT was funded it could do some of this data work. There were questions as to whether it would be possible to make the IHRT inclusive of all centres. Dr Bell felt that clinical data collection component might provide the opportunity for multicentre involvement. He gave an example of a giant cell bone tumor study involving ten centres across Canada. Dr Eisenhauer emphasized the importance of using the data from the 700 patients in the CSG soft tissue sarcoma database to generate ideas for trials, and suggested that the CSG might take a lead internationally on Intergroup trials. The American College of Surgeons is developing a fibromatosis trial and Elizabeth Eisenhauer suggested that this should be proposed as an Intergroup study to NCIC-CTG. There was a proposal that the CSG be more involved in clinical management standards and guidelines. Dr Bramwell expressed reluctance about getting into this area because there are already provincial initiatives which are better funded. Dr. Rajaraman suggested that the CSG might examine and review the Ontario guidelines and perhaps endorse and distribute these to the rest of the country. Dr Bramwell stated that if a CSG web site was established, a link to the Ontario Practice Guidelines Initiative might be the most practical. There was a suggestion that the CSG might be indirectly involved in education and that this could be done by small group meetings at venues such as the NCIC-CTG meeting. Research ideas might come out of smaller meetings with a focused agenda. Dr Bell asked how people were funded to attend the NCIC-CTG meeting and Dr Eisenhauer commented that this was generally by office, ie. site chairs, principal investigators, trial committee members, and by invitation, the number of which given to any one centre depended on the numbers of patients entered onto clinical trials.

The meeting was concluded. Dr Bramwell will write a report to be submitted to the CSG Executive for the NCIC-CTG meeting on 17 April 2000.

Vivien H.C. Bramwell
PhD, MB, BS, FRCP

### PARTICIPANTS LIST

Dr Ben Alman, Hospital for Sick Children, Toronto, ON, Canada
Dr Irene Andrulis, Samuel Lunenfeld Research Institute, Toronto, ON, Canada
Dr Henrik Bauer, Karolinska Hospital, Stockholm, Sweden
Dr Robert Bell, Mount Sinai Hospital, Toronto, ON, Canada
Dr Veronique Benk , Montreal General Hospital, Montreal, QC, Canada (unable to attend because of weather)
Dr Martin Blackstein, Mount Sinai Hospital, Toronto, ON, Canada
Mrs Sophie Boulakia, Toronto, Canada
Dr Vivien Bramwell, London Regional Cancer Centre, London, ON, Canada
Dr Charles Catton, Princess Margaret Hospital, Toronto, ON, Canada
Dr Ann Chambers, London Regional Cancer Centre, London, ON, Canada
Dr Bruce Colwell, Nova Scotia Cancer Centre, Halifax, NS, Canada
Dr Nigel Colterjohn, Hamilton HS Corp-Henderson S, Hamilton, ON, Canada
Dr Jean Couture, Mount Sinai Hospital, Toronto, ON, Canada
Dr Aileen Davis, Mount Sinai Hospital, Toronto, ON, Canada
Dr Chris DeGara, Cross Cancer Institute, Edmonton, AB, Canada
Dr Josee Doyon, Clinique Maisonneuve-Rosemont, Montreal, QC, Canada (unable to attend because of weather)
Dr Pierre Dube, Hop Maisonneuve-Rosemont, Montreal, QC, Canada (unable to attend because of weather)
Dr Elizabeth Eisenhauer, NCIC-CTG, Kingston, ON, Canada
Dr Samy El-Sayed, Cancer Care Manitoba, Winnipeg, MB, Canada
Dr Victor Fornasier, The Wellesley Central Hospital, Toronto, ON, Canada
Dr Marielle Fortier, London Health Sciences Centre, London, ON, Canada
Dr Michael Gross, New Halifax Infirmary, Halifax, NS, Canada
Dr Ab Guha, Toronto Western Hospital, Toronto, ON, Canada
Dr Alex Hammond, London Regional Cancer Centre, London, ON, Canada
Dr Richard Hill, PMH/Ontario Cancer Institute, Toronto, ON, Canada
Dr Ingrid Hings, Montreal General Hospital, Montreal, QC, Canada
Dr Mark Isler, Clinique Maisonneuve-Rosemont, Montreal, QC, Canada
Dr Rita Kandel, Mount Sinai Hospital, Toronto, ON, Canada
Dr Meg Knowling, BC Cancer Agency, Vancouver, BC, Canada
Dr Crystal Mackall, NCI-Pediatric Oncology Branch, Bethesda, Maryland, USA (unable to attend because of weather)
Dr David Malkin, Hospital for Sick Children, Toronto, ON, Canada
Dr Bas Masri, Vancouver General Hospital, Vancouver, BC, Canada
Dr Don Morris, Tom Baker Cancer Centre, Calgary, AB, Canada
Dr John O'Connell, Vancouver General Hospital, Vancouver, BC, Canada
Dr Brian O'Sullivan, Princess Margaret Hospital, Toronto, ON, Canada
Dr Malti Patel, Hamilton Regional Cancer Centre, Hamilton, ON, Canada
Dr Mal Rajaraman, Nova Scotia Cancer Centre, Halifax, NS, Canada
Dr Martin Robinson, Weston Park Hosp NHS Trust, Sheffield, UK
Dr Poul Sorensen, BC Children's Hospital, Vancouver, BC, Canada
Dr Jeremy Squire, OCI/PMH, Toronto, ON, Canada
Dr W.P. Steward, Leicester Royal Infirmary, Leicester, UK
Dr Carol Swallow, Mount Sinai Hospital, Toronto, ON, Canada
Dr Walley Temple, Tom Baker Cancer Centre, Calgary, AB, Canada
Dr Robert Turcotte, Clinique Maisonneuve-Rosemont, Montreal, QC, Canada
Dr Shail Verma, Ottawa General Hospital, Ottawa, ON, Canada
Dr Lorna Weir, BC Cancer Agency-Vancouver CC, Vancouver, BC, Canada
Dr Larry White, Mount Sinai Hospital, Toronto, ON, Canada
Dr Ralph Wong, Cancer Centre Manitoba, Winnipeg, MB, Canada
Dr Jay Wunder, Mount Sinai Hospital, Toronto, ON, Canada
Mr Mark Zuk, London, ON, Canada

